# Remarkable Diversity in the Enzymes Catalyzing the Last Step in Synthesis of the Pimelate Moiety of Biotin

**DOI:** 10.1371/journal.pone.0049440

**Published:** 2012-11-09

**Authors:** Madelyn M. Shapiro, Vandana Chakravartty, John E. Cronan

**Affiliations:** 1 Department of Microbiology, University of Illinois, Urbana, Illinois, United States of America; 2 Department of Biochemistry, University of Illinois, Urbana, Illinois, United States of America; Baylor College of Medicine, United States of America

## Abstract

Biotin synthesis in *Escherichia coli* requires the functions of the *bioH* and *bioC* genes to synthesize the precursor pimelate moiety by use of a modified fatty acid biosynthesis pathway. However, it was previously noted that *bioH* has been replaced with *bioG* or *bioK* within the biotin synthetic gene clusters of other bacteria. We report that each of four BioG proteins from diverse bacteria and two cyanobacterial BioK proteins functionally replace *E. coli* BioH *in vivo*. Moreover, purified BioG proteins have esterase activity against pimeloyl-ACP methyl ester, the physiological substrate of BioH. Two of the BioG proteins block biotin synthesis when highly expressed and these toxic proteins were shown to have more promiscuous substrate specificities than the non-toxic BioG proteins. A postulated BioG-BioC fusion protein was shown to functionally replace both the BioH and BioC functions of *E. coli*. Although the BioH, BioG and BioK esterases catalyze a common reaction, the proteins are evolutionarily distinct.

## Introduction

**Figure 1:**
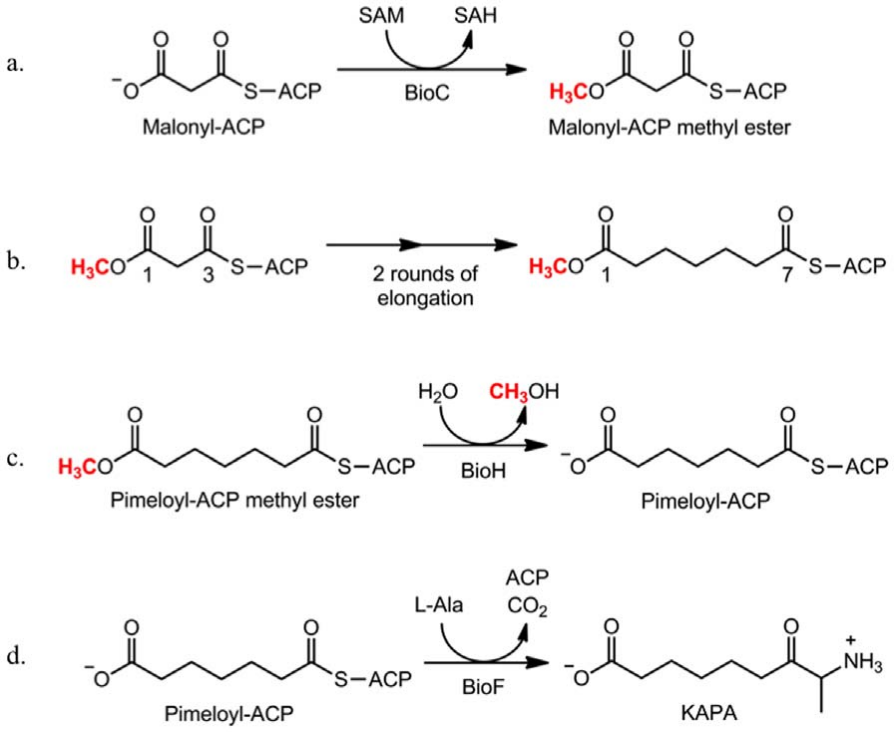
The *E. coli* biotin synthetic pathway. The biotin synthetic pathway is initiated (a) by BioC-catalyzed and S-adenosyl-L-methionine (SAM) mediated methylation of malonyl-ACP. The methyl group is red. The malonyl ACP methyl ester enters the fatty acid synthetic cycle as the primer. (b) Following for two rounds of the fatty acid chain elongation cycle the resulting pimeloyl-ACP methyl ester is then (c) hydrolyzed by BioH to form pimeloyl-ACP which is a substrate for BioF to begin assembly of the biotin rings (d). Abbreviations: SAH, S-adenosyl-homocysteine; AON, 8-amino-7-oxononanoate.

Biotin (vitamin H) is an essential enzyme cofactor required by all three domains of life. It functions as a covalently-bound prosthetic group which mediates the transport of CO_2_ in many vital metabolic carboxylation, decarboxylation and transcarboxylation reactions [1], [2]. Although biotin is an essential cofactor, our knowledge of its biosynthesis remains fragmentary. Labeling studies in *Escherichia coli* suggested that most of the carbon atoms of biotin are derived from pimelic acid, a seven carbon α,ω-dicarboxylic acid [3], [4]. The pathway whereby the pimelate moiety is synthesized was a long-standing puzzle until recent work in *E. coli* showed that it is made by a modification of the fatty acid synthesis pathway that allows synthesis of dicarboxylic fatty acids by a mechanism reminiscent of that proposed in 1963 [5]. Two enzymes, BioC and BioH, hijack a fraction of the fatty acid biosynthetic capacity to make the pimelate moiety. In this, the first complete biotin synthetic pathway, BioC converts the free carboxyl group of a malonyl thioester to its methyl ester [6]. Methylation cancels the charge of the carboxyl group and provides a methyl carbon to mimic the methyl ends of normal acyl chains to give a species approximating the substrates normally accepted by the fatty acid synthetic enzymes (Figure 1). Two cycles of the standard elongation-reduction-dehydration-reduction cycle of fatty acid synthesis results in the acyl carrier protein (ACP) thioester of monomethyl pimelate. The methyl ester of this product is then cleaved by BioH to give pimeloyl-ACP which reacts with alanine in the BioF reaction to give the first intermediate of biotin ring assembly. Thus, the methyl ester disguises the biotin synthetic intermediates such that they are accepted as substrates by the fatty acid synthetic pathway [6]. Although carbon chain elongation requires that the carboxyl group of the primer end of the acyl chain be neutralized by a methyl group [6], it must be freed later in the pathway because the carboxyl is required for biotin protein ligase-catalyzed attachment of biotin to its cognate enzyme proteins.

**Figure 2:**
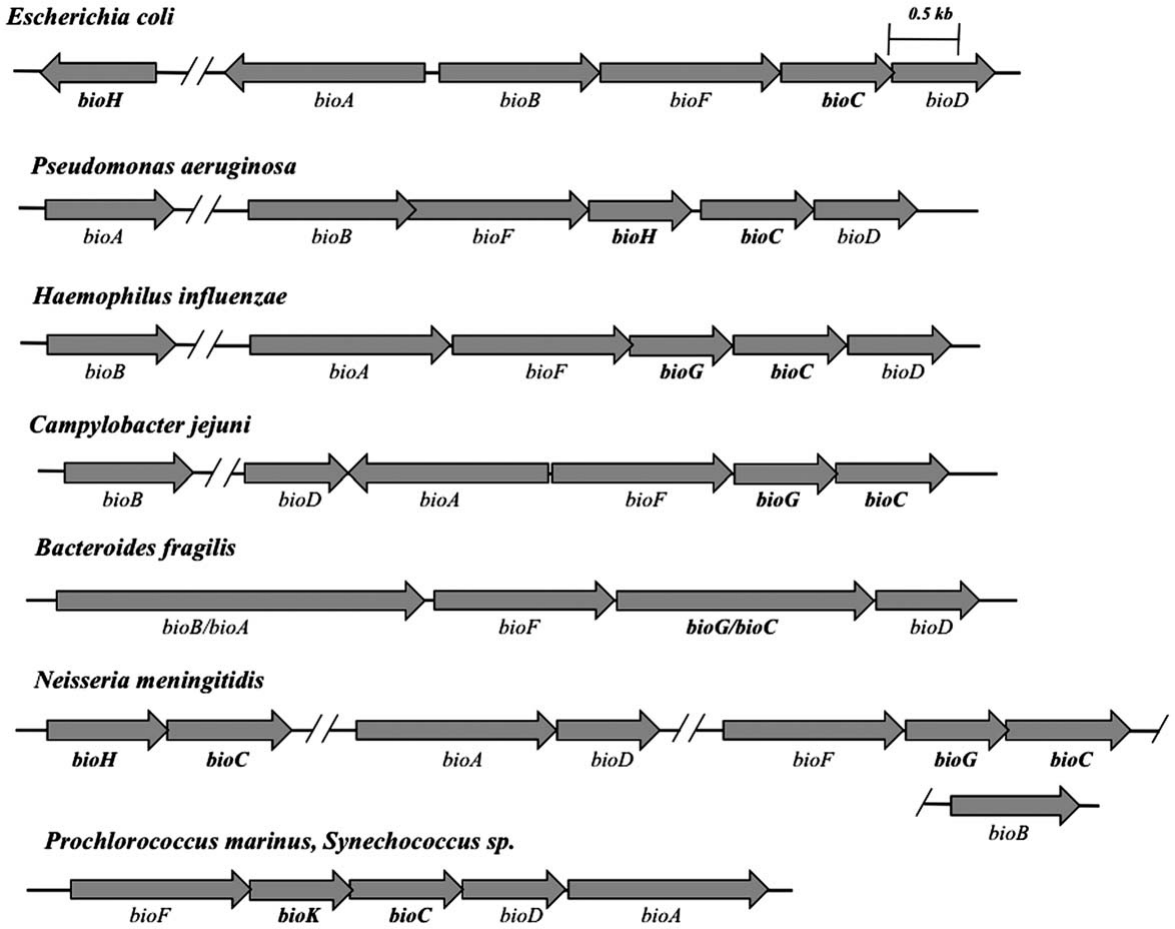
The differing configurations of the biotin genes of diverse bacteria. *E. coli* and *P. aeruginosa* both contain *bioH* but *P. aeruginosa* has *bioH* within its *bio* operon upstream of *bioC* whereas the *E. coli bioH* is located elsewhere on the chromosome. *H. influenzae* and *C. jejuni* have *bioG* within their *bio* operons upstream of *bioC*. *N. meningitidis* has both *bioH* and *bioG* upstream of separate copies of *bioC*. *B. fragilis* encodes a fusion of BioC and BioG. In place of BioG or BioH, most BioC-containing cyanobacteria carrying, such as *Synechococcus spp.* and *P. marinus*, have *bioK* upstream of *bioC*.

In *E. coli* the biotin synthetic genes are located in two distant genome locations. The *bioA*, *bioB*, *bioF*, *bioC* and *bioD* genes are clustered and transcribed by two face-to-face promoters in a bidirectional operon [7]. However *bioH*, the remaining biotin gene, is located far from the *bio* operon (Figure 2) and unlike the other *bio* genes its transcription is not regulated by BirA, the *E. coli* bifunctional repressor-biotin protein ligase [8]–[10]. This gene arrangement is in contrast to those of many other bacteria (e.g., the *Pseudomonadaceae*, *Bacillus cereus*) where *bioH* is located within the biotin operon immediately upstream of *bioC* [11] and is well integrated into the operon (the coding sequences of biotin operon genes often overlap). Thus, the *E. coli bioH* gene may have been more recently acquired than the more “domesticated” *bioH* genes located in *bio* operons. *E. coli* is not the only bacterium in which *bioH* is removed from the *bio* operon. *Yersinia sp.*, *Shewnella sp.*, and *Serratia proteamaculans* share this property, although only the last of these has been shown to functionally replace *E. coli bioH* [12].

Based on bioinformatics analyses Rodionov and coworkers [11] reported that BioH is something of a “wild card” among biotin synthesis enzymes because in some bacteria the gene has been displaced from the biotin operon by other genes (*bioG bioK* and *bioZ*) (Figure 2). These workers proposed that their observation can be explained “either by utilization of different sources for biotin biosynthesis or by nonorthologous displacements of the BioC-linked proteins” [11]. It should also be noted that like BioH, BioG and BioK are upstream of and overlapping with BioC in all of the organisms examined (Figure 2). In our work we furthered and tested the hypotheses of Rodionov and coworkers [11] and found that BioG and BioK are members of the α,β-hydrolase family like BioH and therefore seemed likely to be esterases able to cleave the methyl ester of pimeloyl-ACP whereas BioZ, which is confined to the α-proteobacteria, probably plays a very different biosynthetic role. We report that BioG and BioK proteins of diverse bacteria can replace the BioH function in the *E. coli* biotin synthetic pathway and that several purified BioG proteins cleave the methyl ester of pimeloyl-ACP *in vitro* with varying degrees of specificity.

## Materials and Methods

### Growth media

Genetic manipulations were done in LB broth or agar [13]. Strains grown on M9 minimal medium or agar contained 0.2% arabinose or 0.2% glycerol plus avidin (0.1 U/ml). When supplemented with biotin, 4 nM was the final concentration. The antibiotics used were (µg/ml) sodium ampicillin. 100: kanamycin sulfate; spectinomycin sulfate, 50 and chloramphenicol, 25. The genomic DNAs were obtained from the ATCC.

### Plasmid Constructs

The strains, plasmids and primers used are listed in Table 1. To assemble constructs for the complementation analysis, the *bioG* coding sequences were PCR amplified from the genomic DNAs of *H. influenzae* Rd KW20, *N. meningitidis* MC58, and *C. jejuni* 81–176 [14] were using primers P2 and P7, P3 and P4 and P29 and P30, respectively. The *bioGC* gene was PCR amplified from *B. fragilis* ATCC 25285 genomic DNA using primers P6 and P8. Custom, codon optimized *bioK* genes of *P. marinus* MIT 9211 and *Synechococcus sp.* CC9902 were synthesized with restriction sites added on the vector by IDT, Inc. such both could be amplified using the same primers, P31 and P32. The PCR products of *H. influenzae* and *B. fragilis* were digested with KpnI and HindIII and ligated into pBAD322 digested with the same enzymes to form pMad9 and pMad6, respectively. The PCR products from *N. meningitidis*, *C. jejuni*, *P. marinus* and *Synechococcus sp.* were similarly digested and ligated into pBAD322 [15] to form pMad12, pMad76, pMad77, and pMad78, respectively, except that the enzymes used were NcoI and HindIII for *N. meningitidis* and XbaI and SalI for *C. jejuni bioG* plus both *bioK* genes.

**Table 1:** Bacterial strains, genomic DNAs and plasmids.

Strain	Relevant Characteristics	Reference
BL21(DE3)	*E. coli* B *omp*T *hsd*S_B_ *gal dcm* (DE3)	Invitrogen
Tuner	*ΔlacZY* of BL21(DE3)	Novagen
MG1655	*E. coli* K-12 wild type strain	CGSC
STL24	MG1655 *ΔbioH::FRT*	[6]
STL25	MG1655 *ΔbioC::FRT ΔbioH::FRT*	[6]
STL11	BL21(DE3)/pSTL4	[6]

Plasmid	Relevant characteristics	Primer set	Reference
pBAD322	Amp^R^ arabinose-inducible expression vector		[15]
pET28b+	Kan^R^ T7 expression vector		Novagen
pSTL4	pET28b+ encoding C-term His_6_-*E. coli bioH*		[6]
pMAD6	pBAD322 encoding *B. fragilis bioGC*	P6+P8	This study
pMAD9	pBAD322 encoding *H. influenzae bioG*	P2+P7	This study
pMAD12	pBAD322 encoding *N. meningitids bioG*	P3+P4	This study
pMAD76	pBAD322 encoding *C. jejuni bioG*	P29+P30	This study
pMAD77	pBAD322 encoding *P. marinus bioK*	P31+P32	This study
pMAD78	pBAD322 encoding *Synechococcus bioK*	P31+P32	This study
pMAD23	pET28b+ encoding C-term His_6_-*H. influenzae bioG*	P15+P16	This study
pMAD27	pET28b+ encoding C-term His_6_-*N. meningitidis bioG*	P17+P18	This study
pMAD40	pET28b+ encoding C-term His_6_-*B. fragilis bioGC*	P19+P20	This study
pMAD97	pET28b+ encoding N-term His_6_-*C. jejuni bioG*	P46+P30	This study
pMAD70	pET28b+ encoding C-term His_6_-S65A *H. influenzae bioG*	P52+P53	This study

Primer	Name	Restriction sites	Sequence (5′ - 3′)
P2	HI Rd KW20 bioG rev	HindIII	GAATTAAGCTTAATGATCCCATAATGC
P3	NM MC58 bioG for	NcoI	TTGCCCATGGAAACAAAATTTTACAATC
P4	NM MC58 bioG rev	HindIII	GAATTAAGCTTCAGTGATTCCATAGTGCCG
P6	BF NCTC 9343 bioGC rev	HindIII	GAATTAAGCTTATCTCTTTTTTGC
P7	HI bioG for	KpnI	AAGGTACCATATGAAAACG
P8	BF bioGC for	KpnI	ATGGTACCATGAAACTGAAAAGAC
P15	HI bioG for 2	XbaI	ATATCTAGAAGGAGATATATCAATGAAAACGAAATTTTACGATTAC
P16	HI bioG rev 2	XhoI	GTGCTCGAGATGATCCCATAATGCCGAC
P17	NM bioG for 2	XbaI	ATATCTAGAAGGAGATATATCAATGGAAACAAAATTTTACAATCATC
P18	NM bioG rev 2	XhoI	CTTCTCGAGGTGATTCCATAGTGCCGAC
P19	BF bioGC for 2	XbaI	ATATCTAGAAGGAGATATATCAATGAAACTGAAAAGACTATATC
P20	BF bioGC rev 2	XhoI	GTGCTCGAGTCTCTTTTTTGC
P29	CJ 81–176 bioG for	XbaI	AAGTCTAGAGGAGGTAAAGGGCATGAAAATAAGCAATTTAATTC
P30	CJ 81–176 bioG rev	SalI	TATCTGTCGACTCAAATTTCATCCCAAG
P31	bioK pIDTblue for	XbaI	GCACCTCTAGAAGGAGGTAAATAATG
P32	bioK pIDTblue rev	SalI	CACCGTCGACTTACACTTTATGC
P46	CJ bioG pET N for	NdeI	CACGGTACCATATGAAAATAAGCAATTTAATTC
P52	HIbioG S65A S 2	N/A	CTATCGGCACATCCGTTTGGTGGCGTGGGCGATGGGCGTTTGGGTGGCAG
P53	HIbioG S65A AS 2	N/A	CTGCCACCCAAACGCCCATCGCCCACGCCACCAAACGGATGTGCCGATAG

To assemble constructs for overexpression and purification, the *bioGs* of *H. influenzae*, *N. meningitidis*, and *C. jejuni* were amplified using primers P15 and P16, P17 and P18, and P46 and P30, respectively, whereas *B. fragilis bioGC* was amplified using primers P19 and P20. The *bioG* PCR products of *H. influenzae* and *N. meningitidis* and *bioGC* from *B. fragilis* were digested with XbaI and XhoI and ligated into pET28b+ digested with the same enzymes to give pMad23, pMad27 and pMad40, respectively, in which the putative esterase genes encode C-terminal hexahistidine-tagged proteins. Similarly the *bioG* PCR product of *C. jejuni* was digested with NdeI and SalI and ligated into pET28b+ cut with the same enzymes to give pMad97 which encodes an N-terminal hexahistidine-tagged protein.

Site directed mutagenesis was done using the QuickChange site-directed mutagenesis method (Stratagene) with primers P52 and P53 to alter the putative active site residues of *H. influenzae* BioG (pMad23) (Table 1). *Pfu* polymerase was the PCR polymerase. The PCR reaction product (pMad70) was ethanol precipitated and then introduced into strain DH5α by chemical transformation. The mutations were verified by sequencing (ACGT, Inc). The plasmids pMad9, pMad12, pMad76, pMad77, and pMAD78 were transformed into *E. coli* strain STL24 (*ΔbioH*) whereas pMad6 was transformed into strain STL25 (*ΔbioC ΔbioH*). The overexpression constructs, pMad23, pMad27, pMad40, pMad97, and pMad70 were each transformed into BL21(DE3) and Tuner (Novagen).

### High level expression and purification of BioH and BioG

The protocol to purify BioH and BioG was adapted from that used to purify *E. coli* BioH [6]. Strains BL21(DE3) or Tuner (Novagen) carrying a pET28b+ plasmid encoding a BioG or *E. coli* BioH were grown to OD_600_ of 1 in LB-kanamycin medium at 37°C and overexpression was induced by addition of 1 mM IPTG. The gene products encoded by pMad23 and pMad40 were soluble when transformed into BL21(DE3) and grown at 37°C for 3 h or 21°C for 16 h, respectively, whereas the gene products encoded by pMad27, pMad97, and pMad70 were soluble in Tuner after incubation at 21°C for 16 h. The cells were harvested by centrifugation and the cell pellets were washed with M9 salts and stored at −20°C.

All protein manipulations were done at 4°C or on ice. The cell pellets were resuspended in lysis buffer containing 50 mM 3-(N-morpholino)propanesulfonic acid (MOPS), 10% glycerol, 5 mM 2-mercaptoethanol, 0.5 M NaCl and 20 mM imidazole (pH 7.5). The suspension was passed twice through a French pressure cell then centrifuged 15,000 RPM for 1 h to isolate the soluble extract which was mixed for 30 min with Ni-NTA resin (Qiagen) that had been previously equilibrated in lysis buffer. The resin was then washed twice with lysis buffer and twice with wash buffer (lysis buffer containing 40 mM imidazole). After resuspension in wash buffer the resin was loaded into a column. After flow through of the wash buffer was complete the column was eluted with lysis buffer containing 180 mM imidazole and fractions were collected. Following purity estimation by SDS-PAGE, the fractions were pooled and dialyzed overnight using Slide-A-Lyzer cassettes (Pierce Chemical) against a buffer of 25 mM MOPS, 10% glycerol, 1 mM tris(2-carboxyethyl)phosphine (TCEP) and 0.2 M NaCl (pH 7.5). *E. coli* BioH, *H. influenzae* BioG, and *B. fragilis* BioGC were concentrated using Millipore centrifugal concentrators (10,000 MWCO). The proteins were then flash frozen and stored at −80°C.

The hexahistidine-tagged BioG and BioGC proteins were dried under vacuum, and the mass was analyzed by MALDI-TOF/ESI mass spectrometry at University of Illinois, School of Chemical Sciences Mass Spectrometry Laboratory. Size exclusion chromatography was done on a Superdex 200 analytical size exclusion column calibrated with protein standards from Bio-Rad. The BioG proteins eluted between the chicken ovalbumin (44 kDa) and equine myoglobin (17 kDa) protein standards indicating monomeric proteins. Given that both partners in the BioGC fusion protein are monomeric, BioGC was expected be monomeric and this was the case.

### Esterase activity assays

Each reaction contained 50 mM Tris-HCl (pH 7.0), 5% glycerol, 40 µM pimeloyl-ACP methyl ester (or a shorter or longer homologue) and 5 µg/ml of a putative esterase. The mixtures were incubated for 1 h at 37°C and the products were run on a 20% polyacrylamide gel with 2.5 M urea at 130 V for 3 h. ACP was expressed and purified as previously described [16]. The mono-methyl esters of the dicarboxylic acids were obtained and converted to ACP thioesters using acyl-ACP synthetase as previously described [6].

## Results

### BioG, BioK and BioH share conserved residues characteristic of esterase activity

**Figure 3:**
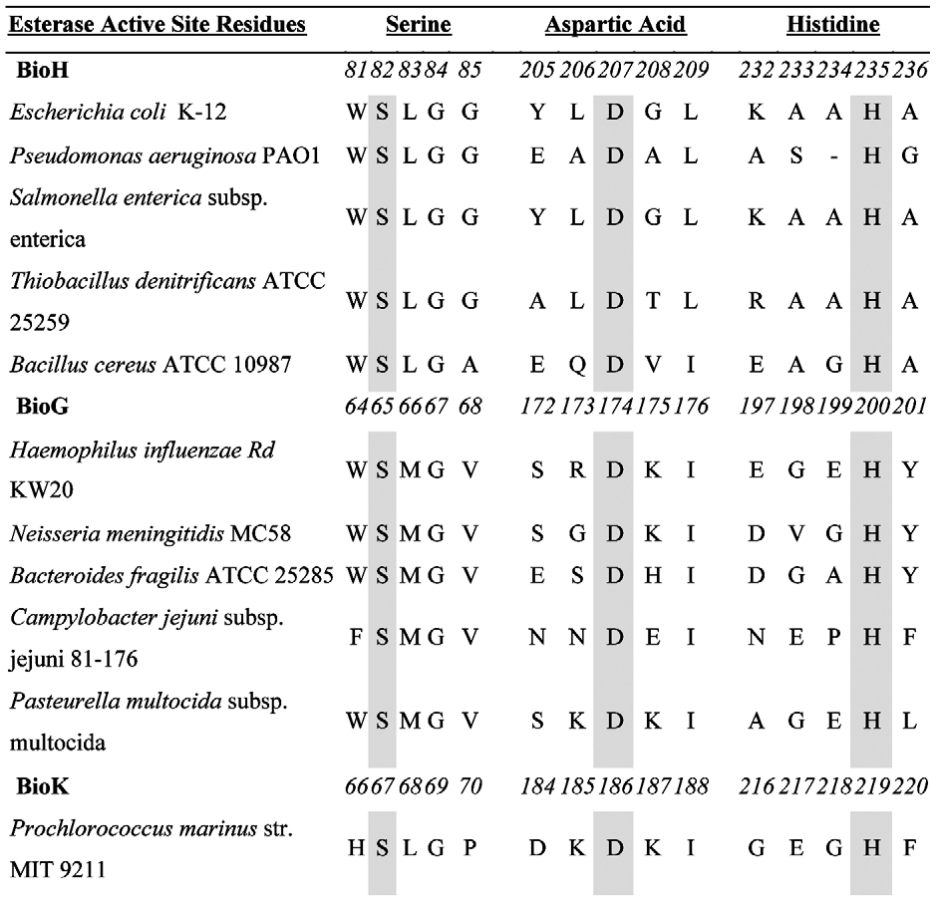
Sequence alignments putative biotin synthetic esterases show conserved catalytic triad residues. Homologues of BioH, BioG and another putative isozymes, BioK, were obtained from the SEED database (http://theseed.uchicago.edu/FIG/index.cgi). Shown are some of the sequences from a MUSCLE [18] alignment with a −1 extension gap penalty. The putative catalytic sites are shaded in yellow. The residue numbers (given in Italics) are those of *E. coli* BioH, *H. influenzae* BioG and *P. marinus* BioK.

We performed bioinformatics analyses of sixteen BioGs and seventeen BioKs using MUSCLE [17], [18] and found that these proteins had the hallmarks of α,β-hydrolases, most notably all contain the aspartic acid, histidine and putative catalytic serine residues characteristic of esterases [19] that aligned with those of BioH proteins (Figure 3). Conservation of the catalytic triad regions among a number of BioG, BioK and BioH sequences suggested that BioG and BioK proteins could be capable of functioning in place of *E. coli* BioH. Note that an *E. coli* BioH crystal structure was obtained several years ago [20] that demonstrated the catalytic triad and identified serine-82 as the nucleophile. More recently, the structure of a BioH-methyl-pimeloyl-ACP complex was determined that allowed demonstration that BioH action prevents elongation of the pimeloyl moiety to a physiologically useless product [21].

### BioG and BioK both replace *E. coli* BioH *in vivo*

All known *bioG* and *bioK* genes are found immediately upstream of *bioC* where the operon-sited *bioH* genes are found [11]. Moreover, *Bacteriodes fragilis* encodes a protein that appears to have BioG fused to BioC [11]. Given these genomic contexts plus our identification of the proteins as putative α,β-hydrolases we tested if expression the *bioG* genes of several diverse bacteria and the *bioK* genes of two cyanobacteria could complement the biotin auxotrophy of an *E. coli* strain carrying a deletion of *bioH* (*ΔbioH*) and thereby allow growth in the absence of biotin. The genes tested were based on several criteria. *H. influenzae bioG* was chosen due to the fairly close evolutionary relationship of this bacterium with *E. coli* whereas *N. meningitidis bioG* was chosen because its genome also contains a *bioH bioC* cluster in addition to the *bioG bioC* cluster. *B. fragilis bioG* was chosen because its coding sequence is fused to that of *bioC*. The *C. jejuni bioG* was chosen because the protein shares only 27% and 24% sequence identity with the BioGs of *H. influenzae* and *N. meningitides* (which are 84% identical to one another). The two cyanobacterial *bioK* genes, those of *P. marinus* MIT-9211 and *Synechococcus sp*. CC9902 were chosen because cyanobacterial proteins tend to have little sequence similarity and these two proteins share only 35% sequence identity.

**Figure 4:**
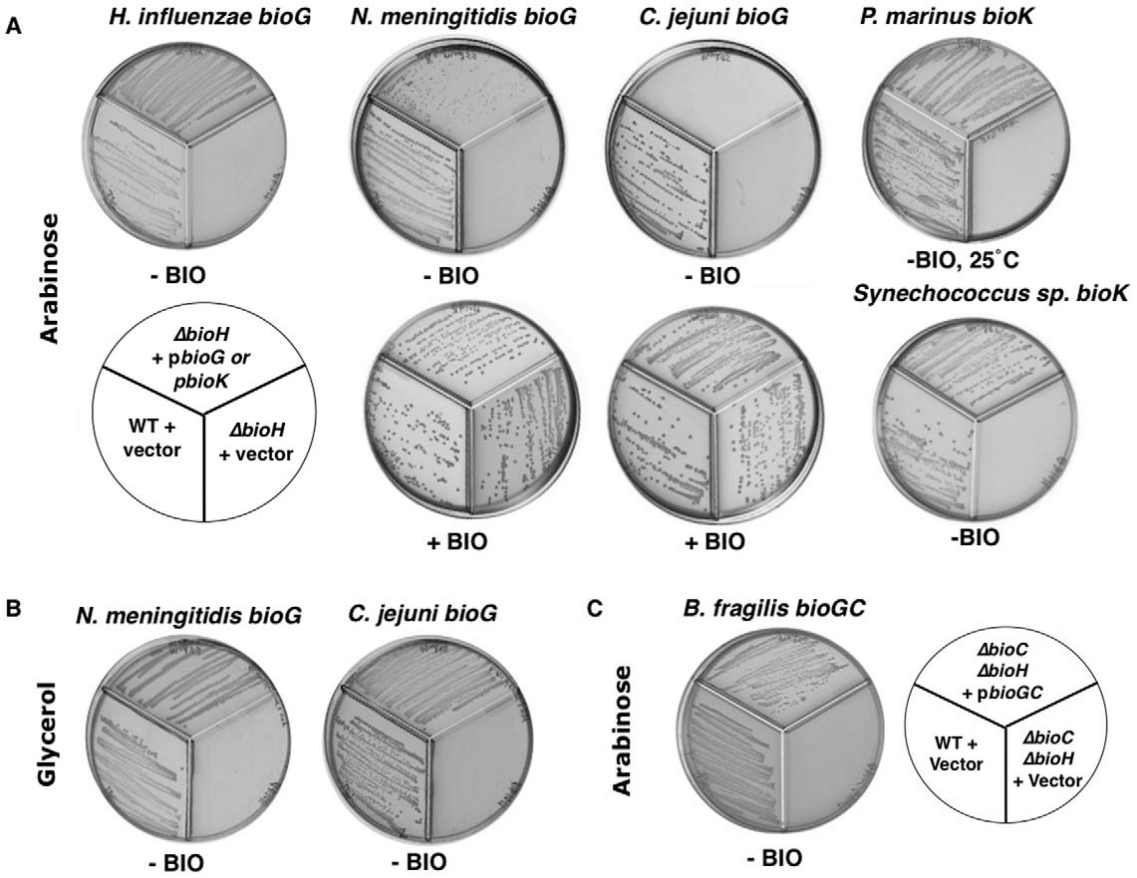
Expression of the *bioG* and *bioK* genes in *E. coli* replaces *bioH* function *in vivo*. *E. coli* strain STL24 (*ΔbioH*) was transformed with derivative of pBAD322 carrying various *bioG* or *bioK* genes. The transformants were streaked on M9 agar plates in the pattern shown on the plate diagram containing the carbon source shown in either the presence or absence of biotin (bio). All plates were incubated at 37°C except those expressing *P. marinus bioK* which were incubated at 25°C. To prevent cross-feeding plates divided into three zones by plastic walls were used. **Panel A.** Arabinose as carbon source, STL24 *ΔbioH* transformed with pBAD322 carrying no insert (lower left third), expressing *bioG* or *bioK* (top third of each plate) and the wild type strain transformed with pBAD322 (lower right of each plate). **Panel B.** The inoculation pattern was the same as Panel A and glycerol was the carbon source in place of arabinose. **Panel C.** The streaking pattern is given by the plate diagram. Arabinose was the carbon source and the test strain was *E. coli* strain STL25 (*ΔbioCΔbioH*) transformed with pBAD322 carrying no insert (lower left third), *bioGC* (top third) or the wild type strain transformed with the vector pBAD322 (lower right third).

To test the function of these genes in the *E. coli* biotin synthetic pathway, pBAD322 plasmid derivatives carrying either *bioG* or *bioK* were transformed into a *ΔbioH* derivative of *E. coli* strain MG1655 and the transformants were streaked onto M9 minimal media lacking biotin that contained either 0.2% arabinose (the inducer of the *araBAD* promoter) or 0.2% glycerol (which gives basal expression) as sole carbon source (Figure 4).

In the absence of biotin and the presence of arabinose the *E. coli ΔbioH* strain expressing *H. influenzae bioG* grew similarly to the wild type strain whereas the *ΔbioH* strain carrying the empty vector showed no growth (Figure 4A). On glycerol, which allows only basal expression from the *araBAD* promoter, growth also proceeded but more slowly. In contrast the *N. meningitidis* and *C. jejuni bioG* genes, respectively, showed poor and no growth of the *E. coli ΔbioH* strain in the presence of arabinose but robust growth occurred when biotin was added (Figure 4A). When glycerol was the carbon source both *bioG* genes supported growth in the absence of biotin (Figure 4B). These growth data indicate that the toxicity of arabinose induction is limited to the biotin synthetic pathway.

The *bioK* genes of *Synechococcus* and *P. marinus* also allowed growth of the *E. coli ΔbioH* strain in the absence of biotin, but only upon arabinose induction (Figure 4A). However, the growth supported by *P. marinus bioK* occurred only at a low growth temperature (25°C) and required four days of incubation (Figure 4B).

To test function of the putative bifunctional enzyme encoded by the *B. fragilis bioGC* gene the plasmid was transformed into a *ΔbioH ΔbioC* doubly deleted derivative of *E. coli* strain MG1655 and streaked on M9 agar containing 0.2% arabinose and lacking biotin. This strain showed strong growth whereas the strain transformed with the empty pBAD322 vector failed to grow (Figure 4C). Thus the *B. fragilis* gene replaced the functions of two *E. coli bio* genes, *bioC* and *bioH*, indicating that both the *bioG* and *bioC* domains of the protein are functional.

Although the growth requirements vary, these data all indicate that BioG and BioK proteins of diverse sequence functionally replace *E. coli* BioH. The complementation of *bioH* by *bioG* and *bioK* also indicates that like BioH, their functions are likely interrelated with that of BioC as implied by the functional fusion of the *B. fragilis* BioC and BioG domains. In addition, the *in vivo* experiments suggest that non-specific hydrolysis of biotin intermediates can occur.

### BioG and BioGC recognize a biotin precursor *in vitro*

**Figure 5:**
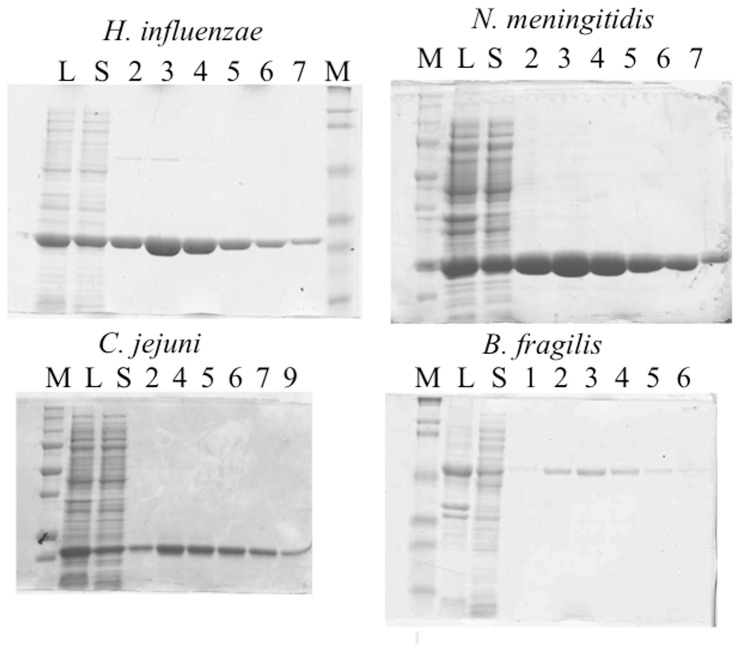
Purification of His_6_-tagged BioG proteins. Samples (10 µl) of each eluted fraction were analyzed by electrophoresis on 10% SDS-polyacrylamide gels. The lysate and soluble fractions are given in lanes L and S, respectively. The protein was eluted from the Ni-NTA column with a buffer containing 200 mM imidazole. The fractions shown were pooled and dialyzed as described in Experimental Procedures. Low range molecular weight markers are shown in lanes marked *M.*

Although the complementation assay and bioinformatics studies indicated that, like BioH, BioG and BioK function as esterases, further studies to characterize these enzymes required *in vitro* studies. Constructs encoding hexahistidine-tagged versions of the BioG and BioGC proteins were expressed and the purified proteins were readily obtained by Ni^2+^-chelate chromatography (Figure 5). The purified proteins were analyzed by both MALDI mass spectroscopy and size exclusion chromatography (Table 2). Unfortunately, this was not the case for the BioK proteins. Both BioK proteins invariably formed insoluble inclusion bodies under a wide variety of expression conditions and thus no active proteins were obtained. The activities of the BioG proteins were determined using a gel electrophoretic mobility shift assay [16]. ACP is a dynamic protein, which attains a large effective radius in this partially denaturing gel system. The protein structure is stabilized against denaturation by acyl chains attached to the ACP prosthetic group with the degree of stabilization depending on the length and polarity of the acyl chain [16].

**Table 2:** Properties of the purified hexahistidine-tagged BioG and BioGC proteins.

Hexa-His tagged Protein	Molecular weight^a^		Solution structure b
	Calculated	Determined	
*H. influenzae* BioG	26265.7	26389.6	monomer
*N. meningitidis* BioG	25965.3	26202.6	monomer
*C. jejuni* BioG	25687.5	25659.7	monomer
*B. fragilis* BioGC	56836.7	56424.8	monomer

a Molecular weight was determined by MALDI-mass spectrometry accurate to within ∼1% except BioGC which was determined by electrospray mass spectrometry. The N-terminal sequence of the N-terminally hexahistidine-tagged *C. jejuni* BioG is Met-Gly-Ser and thus as expected from the specificity of *E. coli* methionine aminopeptidase {Xiao, 2010 #7} the protein lacks the N-terminal methionine residue. The calculated value is for the species lacking methionine.

b Solution structures were assayed by size exclusion chromatography. Each of the BioG proteins eluted between bovine erythrocyte carbonic anhydrase (29 kDa) and horse myoglobin (17 kDa) standards indicating monomeric proteins. The BioGC protein eluted between chicken ovalbumin (44 kDa) and bovine serum albumin (66 kDa) indicating that the protein is a monomer in solution. Note that upon concentration for application to the size exclusion column *B. fragilis* BioGC formed aggregates although an appreciable fraction of the protein eluted at the volume expected for a monomeric protein (between bovine serum albumin and there was no apparent peak where dimer would elute. The fast eluting peaks were identified as BioGC aggregates by SDS-PAGE.

**Figure 6:**
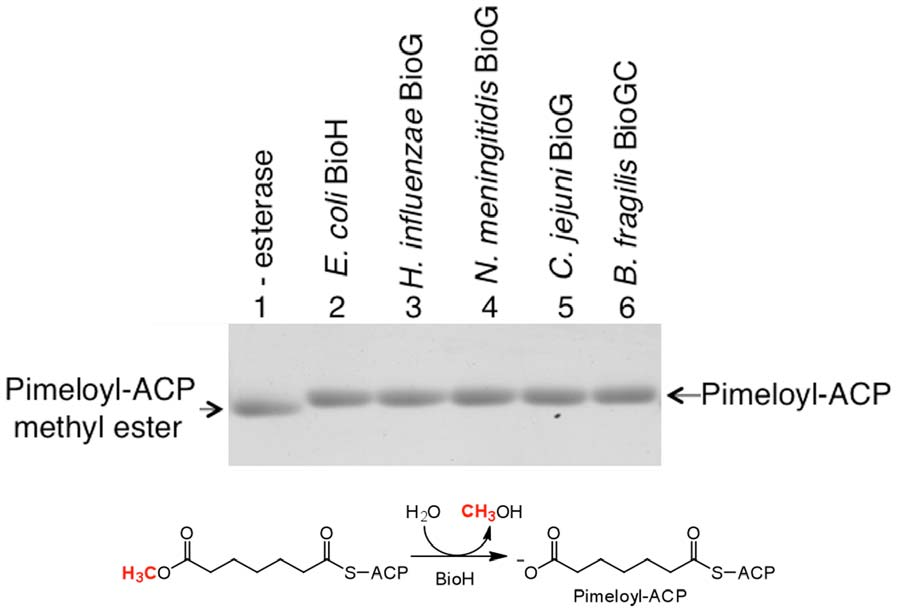
The BioG proteins cleave the ester group of pimeloyl-ACP methyl ester. The reaction mixtures containing pimeloyl-ACP methyl ester were mixed with purified BioH as a positive control or a purified BioG from one of four different bacteria as shown. Lane 1 lacked enzyme added. Following incubation for 1 h 10 µl of the reaction mixture was analyzed by electrophoresis on a 20% polyacrylamide gel containing 2.5 M urea.

The BioG and BioGC proteins were assayed for conversion of pimeloyl-ACP methyl ester, the physiological BioH substrate, to pimeloyl-ACP. As reported previously the reaction mixture containing BioH gave a band of lower mobility indicating hydrolysis of the ester moiety (Figure 6) This is because the new charged ω-carboxyl group plus loss of the hydrophobic methyl ester destabilized the hydrophobic ACP acyl chain binding cleft causing the ACP moiety to expand. The BioG proteins of *H. influenzae*, *N. meningitidis*, and *C. jejuni* as well as the *B. fragilis* BioGC fusion protein hydrolyzed pimeloyl-ACP methyl ester (Figure 5, Lanes 4–6), which shows that under these conditions, the BioGs recognize and hydrolyze the same substrate as *E. coli* BioH. Hence, these data are in excellent accord with the *in vivo* complementation data. Note that this assay has been validated by mass spectroscopy [6].

### *H. influenzae* BioG is a serine esterase

**Figure 7:**
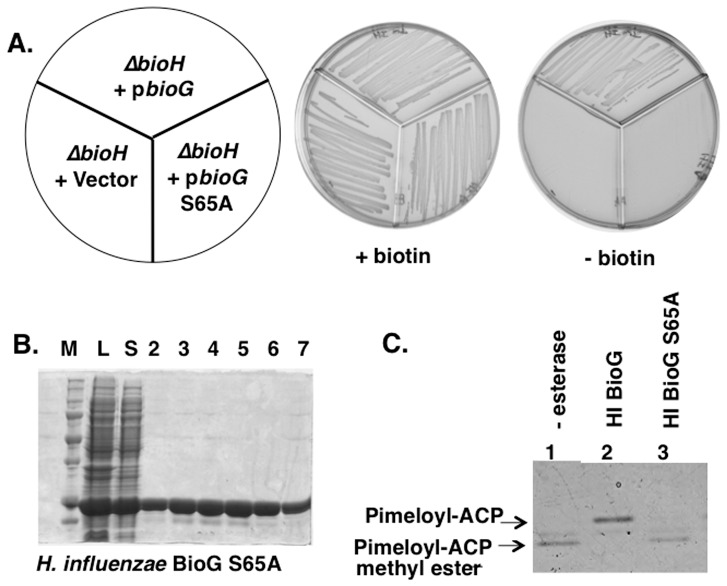
Loss of BioG function upon substitution of the putative active site serine with alanine. **Panel A.** *E. coli* strain STL24 (*ΔbioH*) was transformed with plasmids encoding *H. influenzae* BioG S65A (right), wild type *H. influenzae* BioG domain (top) or the empty pET28b+ vector (left). The transformants were streaked onto M9 plates containing 0.2% glucose. **Panel B** Purification of the S65A BioG. Eluted fractions (10 µl) were analyzed by electrophoresis on a 10% SDS-polyacrylamide gel. The lysate and soluble fractions are shown in lanes L and S, respectively. Panel C. The *H. influenzae* BioG S65A protein was assayed for esterase activity as in Figure 6.

Prior work had shown that substitution of alanine for the putative serine nucleophile of *E. coli* BioH abolished both the *in vivo* and *in vitro* activity of the protein [6]. To test if *H. influenzae* BioG functioned similarly we constructed an S65A derivative of the protein in the pET28b+ expression vector. Based on prior data we expected that the repressed level of expression of wild type *H. influenzae* BioG from the pET28b+ promoter would be sufficient to allow growth of the *E. coli ΔbioH* strain in the absence of biotin. Indeed robust growth was observed (Figure 7A). In contrast the plasmid that encoded the BioG S65A protein failed to allow growth under these conditions. Upon biotin supplementation all strains grew. However, the lack of complementation by BioG S65A could have been the result of inclusion body formation by the mutant protein. This was not the case. The mutant protein was indistinguishable from the wild type BioG protein in that it was readily expressed in soluble form and purified (Figure 7B). However, in contrast to the wild type BioG protein, the BioG S65A protein had no detectable activity in the pimeloyl-ACP methyl ester cleavage assay (Figure 7C). Taken together with the *in vivo* data, it is clear that *H. influenzae* BioG, like *E. coli* BioH, is a serine esterase.

### The BioG proteins have differing degrees of substrate promiscuity

**Figure 8:**
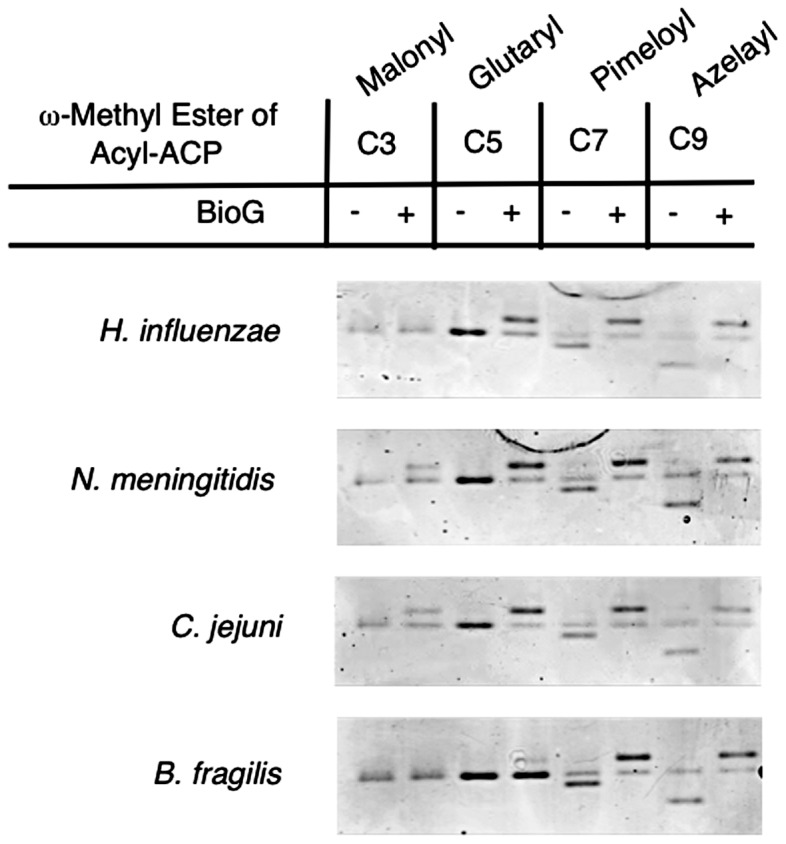
Assay of the purified BioG on shorter and longer analogues of pimeloyl-ACP methyl ester. Reaction mixtures each containing an acyl-ACP methyl ester were either left untreated (−) or treated (+) with one of the BioG proteins as described in Materials and Methods.

In the complementation experiments presented above some of the *bioG* genes required induction of expression by arabinose for robust growth in the absence of biotin whereas others grew well without induction (glycerol as carbon source), but grew very poorly in the presence of arabinose. Moreover, addition of biotin to the arabinose plates allowed normal growth. These results indicated that toxicity was specific to the biotin synthetic pathway. The most straightforward interpretation is that the toxic BioGs are either less specific or are produced at higher levels than the non-toxic proteins and the excesss activity aborts biotin synthesis. We favor that former possibility because toxic and nontoxic BioG proteins showed similar levels of expression in extracts prepared for protein purification (Figure 5). The most plausible biotin pathway target for the toxic BioG would be the short intermediates of pimeloyl moiety synthesis. We assayed hydrolysis of two such intermediates, the ACP thioesters of malonate methyl ester and glutarate methyl ester. Gel mobility shift assays with the four BioG proteins indicate that the two toxic proteins were more promiscuous in their substrate cleavage (Figure 8). When assayed on the physiological C7 substrate and the non-physiological C9 substrate, the four enzymes had comparable activities. However, both of the toxic BioGs, those of *C. jejuni* and *N. meningitides*, cleaved both the C3 and C5 substrates whereas neither of the non-toxic BioGs *H. influenzae* and *B. fragilis* cleaved the C3 substrate. *H. influenzae* BioG cleaved the C5 substrate whereas *B. fragilis* BioG was the least promiscuous of the four enzymes in that it had only trace activity on the C5 substrate (note that the effective BioG concentration was half that of the other enzymes due to the greater size of the fusion protein). These results show that each of these BioGs are capable of hydrolyzing substrates other than the C7 substrate required in biotin synthesis as is the case of *E. coli* BioH which slowly cleaves the C5 acyl-ACP methyl ester moiety [6]. It should be noted that assay by gel mobility shift is not suited for kinetic determinations because in order to observe shifted bands at low substrate concentrations, hydrolysis of a major fraction of the substrate is required and thus the reaction kinetics would be progressively altered during the assays.

### The BioH, BioG and BioK esterases fall into distinct clades

Although BioH, BioG, BioK recognize the same substrate and share the same catalytic residues, their overall sequence identity is low. To examine the phylogenetic relationships of these proteins we first entered a few BioH, BioG and BioK sequences into the Pfam database [22] which placed the esterases into different protein families within the same clan (CL0028). BioHs were members of the α,β -hydrolase 6 family, BioGs the DUF452 family and BioKs are members of the α,β-hydrolase 5 family.

**Figure 9:**
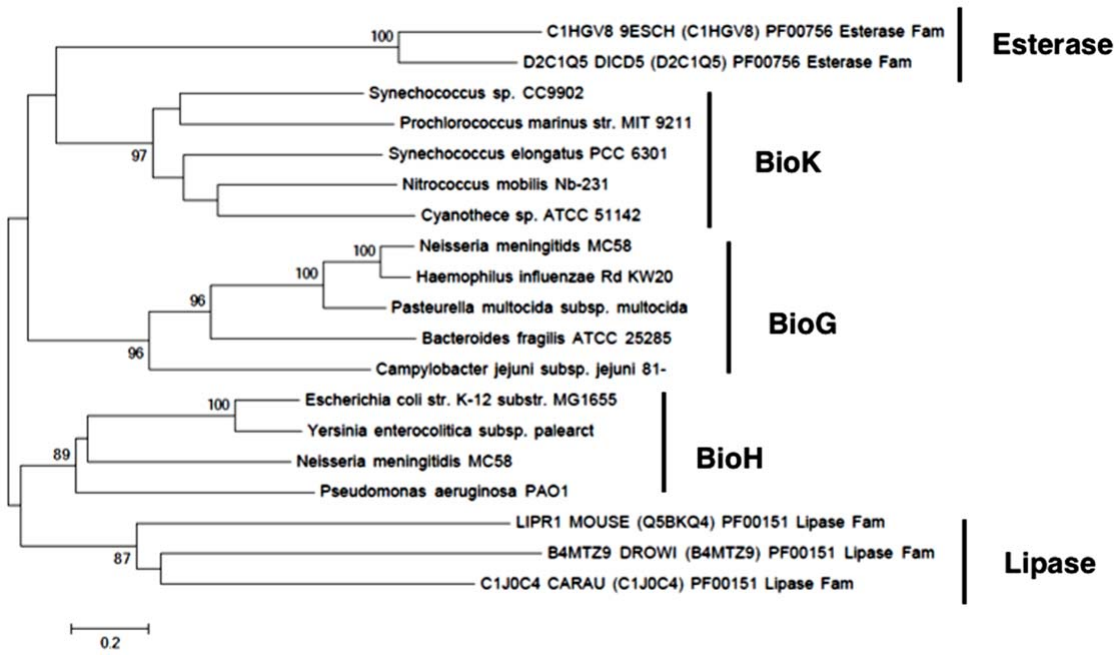
BioH, BioG and BioK are evolutionarily distinct. The evolutionary relationship between sequences from several α,β-hydrolase families was inferred using the Mega5 [32]. Sequences from other families α,β-hydrolases were obtained from the Pfam database [22]. The bootstrap percentage values for 1000 replicates are shown next to the branches. The optimal tree is drawn to scale, with branch lengths in the same units as those of the evolutionary distances (the number of amino acid residue substitutions per site). The scale represents a 50% difference in compared residues per length. The analysis involved 23 amino acid sequences. All positions containing gaps and missing data were eliminated. The final dataset had a total of 148 positions. Bootstrap values lower than 80% are not shown.

To examine the evolutionary distances of BioG, BioH, and BioK a minimum evolution phylogenetic tree was constructed from five BioGs, four BioHs and five BioKs both relative to one other and to outlier sequences from two other families of the same clan, two bacterial S-formylglutathione hydrolases (esterase family) and three eukaryotic lipases (lipase family) (Figure 9). The proteins grouped into five clades, as expected, with relatively high bootstrap values for the nodes linking all of the genes within each clade (96% for BioG, 97% for BioK, 89% for BioH, 87% for the lipases, and 100% for the esterases (Figure 7). This shows that despite the biotin synthesis proteins sharing the same biological function, each had followed its own evolutionary path as seen for the outlier esterases of different biological functions. However, conclusions regarding the relative evolutionary distances of each of the clades from one another cannot be drawn because the node positions between the clades show little bootstrap support.

The ability to determine evolutionary distances between each of the sequences within a biotin synthetic esterase clade vary, however, some distances can be inferred. In the case of BioK, the 97% bootstrap value suggest that the *P. marinus* and *Synechococcus sp* BioKs diverged from a different ancestor than did the other BioKs, however, there is little bootstrap support for the position of the nodes linking each of the BioK sequences to one another. However this shows that even a protein sequence within a clade can follow an evolutionary path distinct from another protein within that clade. In the case of BioH, there is strong evidence that *E. coli* and *Y. enterocolitica* BioHs are more closely related to one another than to *P. aeruginosa* BioH (100% and 89% bootstrap support, respectively), however, there was insufficient bootstrap support to assess the distance of *N. meningitidis* BioH to any of the other BioHs. In contrast, there is strong bootstrap support for the relative distances of all of the BioGs. The distance between the BioGs from *N. meningitidis* and *H. influenzae* are close (100% bootstrap support) compared to the other BioGs. The evolutionary distance from *B. fragilis* BioG to *H. influenzae* and *N. meningitidis* BioG is longer (96% bootstrap support) whereas *C. jejuni* BioG is most distant from any of the other BioG genes (96% bootstrap support). These results show that within both the BioG and BioH clades, the evolutionary distances between sequences can vary greatly even if those sequences share an evolutionary path. Despite the wide variation in evolutionary distances between all of the BioG, BioK, and BioH proteins, the *in vivo* results show that each protein is capable of performing the same hydrolytic function on the C7 acyl-ACP methyl ester moiety. However, considering that supposedly related BioGs (from *H. influenzae* and *N. meningitidis*) have different enzymatic activities under the same conditions, it shows that that even BioGs having similar sequences can have enzyme activities that differ more than two BioGs separated by a greater evolutionary distance (*N. meningitidis* and *C. jejuni*).

## Discussion

Given the strong conservation of the other biotin synthetic enzymes across biology, the diversity of proteins that catalyze cleavage of pimeloyl-ACP methyl ester is striking. None of these enzymes appear to have been newly evolved or acquired because their coding sequences often overlap with both the downstream *bioC* coding sequence and the upstream *bioF* coding sequence (Figure 2, Table 3). Therefore, the esterase genes seem well integrated into their respective operons. Indeed, the translational coupling imparted by overlapping genes should result in a set ratio of esterase activity to that of BioC and BioF which seems important because high level expression of certain BioG proteins results in loss of the ability to replace *E. coli* BioH seen upon moderate expression (Figure 4). Other workers have reported that overproduction of *E. coli* BioH compromises *E. coli* biotin synthesis [10]. As noted above some BioH proteins, notably that of *E. coli*, are not encoded within a biotin synthetic operon, but elsewhere on the genome. Such freestanding genes are not readily identified because bacterial genomes encode many esterases (*E. coli* has at least 15) [23]. Indeed, the *E.* coli *bioH* gene was discovered only when deletion analysis of a neighboring gene cluster engendered a biotin requirement [24]. Hence, there may well be unrecognized examples of BioG and BioK genes located outside biotin gene clusters. Freestanding *bioK* genes would seem particularly difficult to recognize because cyanobacterial proteins have little sequence similarity even among bacteria thought to be closely related. One possible explanation for the diversity of the esterases of biotin synthesis relative to the rather strict conservation seen in the biotin ring formation enzymes is that ester hydrolysis is a simple reaction whereas ring formation requires much more complex chemistry. Indeed, collaborative work from this laboratory has shown that a *P. aeruginosa* PAO1 esterase of unknown function can attain BioH activity by simple amino acid substitutions [25]. Note that like *E. coli* BioH [26] and virtually all other α,β-hydrolases [27], [28], the BioG proteins behave as monomeric proteins in solution (Table 2). Given these data and the finding that *Bacillus cereus* BioC is monomeric [29] the BioGC protein seemed likely to be monomeric and this was the case (Table 2).

**Table 3:** Overlapping Coding Sequences.

	Overlap with (bp)	
Coding Sequence	*bioC*	*bioF*
*Pseudomonas aeruginosa bioH*	8	8
*Bacillus cereus bioH*	35	8
*H. influenzae bioG*	17	0
*N. meningitidis bioG*	13	0
*C. jejuni bioG*	4	4
*P. marinus bioK*	4	4
*Synechococcus bioK*	19	4

A possible caveat to our in vitro data is that we have used *E. coli* ACP rather than the cognate ACP of each of the bacteria. However, since each of the BioGs (as well as the BioKs) replaced BioH function in *E. coli* the BioGs clearly recognize the substrate when attached to *E. coli* ACP. Based on the structure of the complex of BioH with methyl pimeloyl-ACP [21] this may be due to the highly conserved helix II of ACP. The interactions of BioH with the substrate ACP moiety are exclusively with helix II and all of the BioG-containing organisms we tested have ACP helix II sequences very similar to that of *E. coli.*

The *B. fragilis* BioG-BioC fusion protein postulated by Rodionov and coworkers [11] has been expressed and has both of the postulated activities, the protein simultaneously replaces both BioH and BioC in *E. coli* and its BioG activity has been demonstrated *in vitro*. It is interesting that of the *Bacteroides* species of known genome sequence only the three *B. fragilis* genomes encode the fusion protein. The other *Bacteroides* genomes (*B. thetaiotaomicron*, *B. xylanisolvens*, *B. vulgatus*, *B. helcogenes* and *B. salanitronis*) each encode discrete BioG and BioC proteins. The *B. thetaiotaomicron* proteins can be readily aligned (58–60% amino acid residue identity) with the *B. fragilis* fusion protein and the alignments leave only a gap of ten residues between the sequences that align with BioG and BioC. The fusion protein sequence opposite the alignment gap is NLAPAAAASS, a sequence that closely resembles the flexible linker regions that allow inter-subunit communication in enzymes such as pyruvate dehydrogenase and acetyl-CoA carboxylase [30], [31]. Given the gene order of biotin operons formation of the fusion protein can readily be envisioned. However, bifunctional fusion proteins generally catalyze consecutive steps in a pathway (e.g., the bifunctional *E. coli* TrpC and TrpD proteins) whereas the BioC-BioG protein does not; the two reactions are separated by two cycles of fatty acid synthesis. However, if we consider fatty acid synthesis to be an essential cell process that must always be performed (because cells must make membranes), then BioC-catalyzed methylation and BioG catalyzed ester cleavage can be considered consecutive steps.

In conclusion the enzymes that remove the methyl group of methyl-pimeloyl-ACP show a diversity that appears of long standing. Each of the bacteria we studied appear to have acquired a gene that encodes an α,β-hydrolase that performs the required function without disruption of other cellular processes. The gene became integrated into the biotin synthetic operon where it remains a stable entity. Whether the gene encodes BioH, BioG or BioK seems of no consequence and there appears to be little or no selective pressure to favor one gene over another.
